# Toward point-of-care assessment of patient response: a portable tool for rapidly assessing cancer drug efficacy using multifrequency impedance cytometry and supervised machine learning

**DOI:** 10.1038/s41378-019-0073-2

**Published:** 2019-07-15

**Authors:** Karan Ahuja, Gulam M. Rather, Zhongtian Lin, Jianye Sui, Pengfei Xie, Tuan Le, Joseph R. Bertino, Mehdi Javanmard

**Affiliations:** 1 Department of Electrical and Computer Engineering, Rutgers University, New Brunswick, NJ USA; 2 Rutgers Cancer Institute of New Jersey, Rutgers University, New Brunswick, NJ USA

**Keywords:** Electrical and electronic engineering, Sensors

## Abstract

We present a novel method to rapidly assess drug efficacy in targeted cancer therapy, where antineoplastic agents are conjugated to antibodies targeting surface markers on tumor cells. We have fabricated and characterized a device capable of rapidly assessing tumor cell sensitivity to drugs using multifrequency impedance spectroscopy in combination with supervised machine learning for enhanced classification accuracy. Currently commercially available devices for the automated analysis of cell viability are based on staining, which fundamentally limits the subsequent characterization of these cells as well as downstream molecular analysis. Our approach requires as little as 20 μL of volume and avoids staining allowing for further downstream molecular analysis. To the best of our knowledge, this manuscript presents the first comprehensive attempt to using high-dimensional data and supervised machine learning, particularly phase change spectra obtained from multi-frequency impedance cytometry as features for the support vector machine classifier, to assess viability of cells without staining or labelling.

## Microfluidics: Enabling the rapid assessment of cancer drug efficacy

By measuring cells passing through electrodes, this microfluidics device could soon predict patient response to cancer therapy. Mehdi Javanmard and his team from Rutgers University in the United States developed a device that detects whether a cancer cell is alive based on the shift in its electrical properties as it passes through the device’s spectroscopy detection circuit. The team tested their device using cancer cell samples treated with different concentrations of a targeted anti-cancer drug. The device was able to rapidly assess live cell counts up to an accuracy of 95.9%. Future studies will test the device on clinical samples, and it is hoped that this device may one day be used as a convenient assay to pit patient tumor samples against therapies – giving a reliable indicator of response before treatment is administered.

## Introduction

Cancer continues to be one of the leading causes of death worldwide. The primary treatment options for cancer include surgery^1^, chemotherapy^2^, radiation therapy^3^, hormonal therapy^4^, targeted therapy^5^, and palliative care^6^. The choice of therapy mainly depends upon the type and stage of cancer, legal issues, clinical infrastructure, past response rates, and the patient’s health conditions. Chemotherapy is non-specific and results in killing non-targeted cells, which results in many side effects including hair loss and serious gastrointestinal issues. A targeted approach, where only tumor cells are eliminated, with minimal effect on non-tumor cells would result in higher efficacy with minimal side effects. New therapeutic agents as well as diagnostic tools predicting patient response are urgently needed. In this work we present a novel method to rapidly assess drug efficacy in targeted cancer therapy, where antineoplastic agents are conjugated to antibodies targeting surface markers on tumor cells. “Activated” matriptase, a membrane-bound protease, is overexpressed in various epithelial cancer cells, including B-cell lymphoma, multiple myeloma, and epithelial carcinomas^7,8^. Given the importance of matriptase in tumor behavior and its expression on a wide variety of tumor cell types, the targeted delivery of cancer drugs to the tumor site shows great promise for enhancing drug efficacy and minimizing toxicity toward noncancerous cells. Cell sensitivity to drug can be assessed by qualitatively analyzing the surface markers on the tumor cells to determine whether the targeted therapy will be effective or not. Cells sensitive to the anti-matriptase-conjugated drug will undergo apoptosis (cell death), while the cells which are insensitive to the drug will remain alive. We have fabricated and characterized a device capable of rapidly assessing cancer cell viability in response to anti-matriptase-conjugated drugs using multifrequency impedance spectroscopy in combination with machine learning for enhanced classification accuracy, without the need for any staining or labeling of the cells.

The gold standard for automated cell viability analysis is the Vi-Cell instrument developed by Beckman Coulter^®^. It uses the trypan blue dye exclusion method to perform analysis of cell viability. Staining fundamentally limits the subsequent characterization of cells as well as downstream molecular analysis and requires bulky optical instrumentation to assess the viability. In addition, this method requires 0.5–1 mL of sample volume. In addition, various assays are available for measuring different markers which indicate cell death (cytotoxicity assay), the mechanism of cell death, and the quantity of live cells (viability assay)^9,10^. Drug screening platforms utilizing nano and microfluidic channels have been widely explored^11–19^. The use of microfluidic channels can help reduce sample volumes and the cost of reagents. Recently droplet microfluidics has been effectively used for drug screening of cancer cell lines^20^. Apart from microfluidic techniques, optical coherence tomography (OCT) has been used to track cell death^21^. Although OCT is label free and can quantitatively track cell death, the use of optical techniques requires bulky optical instrumentation, making it less compatible with the needs of point-of-care. Screening cells based on their electrical properties can enable label-free assessment of cell viability. The use of dielectropheresis has been vastly explored to assess cell viability, growth, and immuno-reactivity^22,23^. The primary drawback of dielectropheresis is that for each cell, sorting/analysis based on dielectric properties is performed only at a single frequency. The use of multiple frequencies simultaneously can provide a snapshot of a cell’s dielectric properties over a wide range of frequencies, resulting in higher classification accuracy.

Electrical impedance spectroscopy/cytometry enables measuring AC electrical properties of particles in suspension through which the frequency dependent dielectric parameters of the particles can be obtained. The primary advantage of impedance cytometry is that it is label free, and analysis can be performed at a single cell level. The use of bio-impedance measurements can date back to the early 20th century^24,25^ where low and high frequency conductivity of erythrocytes were measured. Since then, there have been many advancements in the field of microfluidic single cell impedance analysis^26^. Microfluidic impedance cytometry has shown promising results in various fields such as analysis and differentiation of leukocytes^27^ and platelets^28^, whole blood cell differentiation^29^, nano-electronic barcoding of particles^30^, tumor cell characterization and classification^31^, and PicoMolar level detection of protein biomarkers^32^, among other fields. In the past, multifrequency impedance cytometry has also been used to analyze membrane potential and viability of *Bacillus megaterium* cells^33^. Although multifrequency impedance cytometry was used, only one frequency (10 MHz) was used to analyze cell viability of *Bacillus megaterium* cells. One frequency may not be sufficient to accurately classify whether a cell is live or dead. We extend the advancements made in impedance cytometry toward a new direction where analysis of cell viability of the cancer cells is based on their impedance response in conjunction with machine learning for enhanced classification accuracy.

A field of computer science that enables computers to learn without explicitly being programmed is machine learning. Evolved from the study of pattern recognition and computational learning theory in artificial intelligence, machine learning plays a pivotal role in the study and construction of algorithms, which learn and make predictions on data. Support vector machines (SVMs) are supervised learning models that have algorithms associated with them to analyze data for classification and regression^34^. SVM’s are efficient in performing nonlinear classification, implicitly mapping their inputs to high-dimensional feature spaces. Machine learning has found wide applications within biology and bioinformatics to make accurate predictions^35–37^. We demonstrate the use of machine learning toward building a portable point-of-care diagnostic tool for assessing patient response to targeted cancer therapy. A patient’s cancer cells are treated with antibody-conjugated drugs, and the proposed impedance cytometer determines the percentage of live and dead tumor cells in the sample. A larger proportion of dead cells in the sample is indicative that the cells express activated matriptase and that the targeted therapy will be effective.

**Fig. 1:**
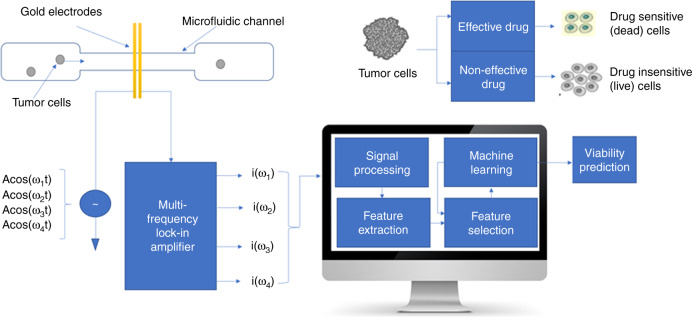
Schematic diagram of the system. Multifrequency impedance cytometry measures the response across a broad range of frequencies for assessment of cellular response to target drug. Live cells and dead cells are assessed using machine learning algorithm to predict their viability

**Fig. 2:**
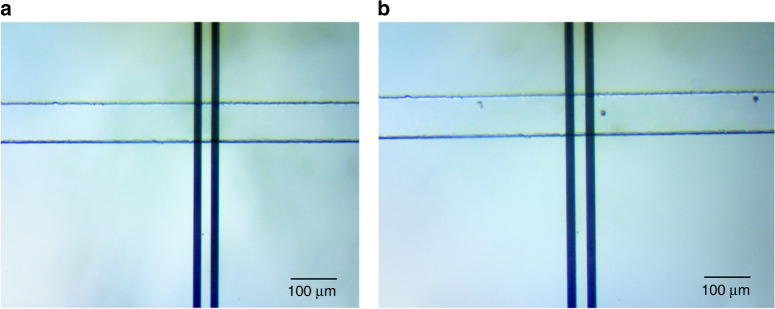
Device micrograph. **a** Microfabricated electrodes at the channel. **b** Cancer cells flowing through the microfluidic channel

Figure 1 illustrates the schematic diagram of the system. This includes a microfluidic channel embedded on a glass wafer with gold electrodes (Fig. 2), a multifrequency lock in amplifier (Zurich Instruments®), and software to record and analyze the data. Figure 2a represents microfabricated electrodes integrated in the channel and Fig. 2b represents cancer cells (T47D) flowing through the microfluidic channel. Impedance cytometry experiments were conducted after the fabrication of devices. In this work we conducted experiments with T47D cancer cells, T47D cancer cells treated with target drug and T47D dead cancer cells. We conducted the impedance cytometry measurements at discrete frequencies ranging from 300 kHz to 30 MHz. For each cell type we performed a series of measurements at four discrete frequencies simultaneously. We always used *f* = 500 kHz as one of the frequencies for each set of measurements.

After the cell culture was performed, and cells were treated with drug for different durations, the cells in the media (RPMI 1640) were centrifuged (290 *g* for 5 min) and suspended in 1× PBS (~400 cells/μL) to perform the impedance cytometry experiments. Impedance cytometry experiments were immediately conducted after their viability was assessed using the Vi-CELL™ Series Cell Viability Analyzer (Beckman Coulter, Carlsbad, CA). We performed impedance cytometry measurements for the following viability percentages: (1) 100% live cells; (2) 100% dead cells; (3) 90% live cells; 82% live cells; 50% live cells. Different viability percentages were obtained by exposing the drug to longer incubation periods.

## Results and discussions

**Fig. 3:**
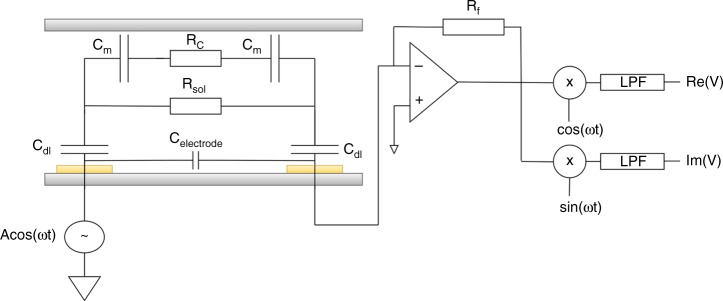
Device circuit model. Equivalent circuit model of the electrode–electrolyte interface in the microchannel along with the readout circuit for measuring changes in resistance across the channel

In our design, we assume an ideal polarizable electrode system with no faradic reactions as we used gold as the electrode material. When a voltage is applied across the two electrodes, it results in a double layer of ions with opposing polarity forming a boundary and acting as a capacitance, which is commonly referred to as the double-layer capacitance. Thus, we use a simplified circuit model (Fig. 3) with a double-layer capacitance (C_dl_) at each electrode in series with the solution resistance (R_s_) in parallel with the coupling capacitance between two electrodes in the cell (C_cell_). Passage of cells trough the pore results in modulation of ionic resistance. The measurements were performed using a lock in amplifier and software to record the data.

The recorded data was then post processed using an algorithm to detrend and denoise the data which helped to analyze the cytometry data with minimal error. After detrending and denoising the data we extracted two significant features from the data: amplitude change and phase change. Amplitude change was termed as change in amplitude level when a cell passes by, which is obtained by finding the difference between the baseline voltage and the peak voltage of a cell passing by. This change in amplitude was calculated for each single cell passing by with respect to its baseline for all the frequencies at which measurements were conducted. Phase change was termed as the change in angular position of the excitation frequency when a cell passes by. This was calculated from the real and imaginary data points obtained from the data. Again, change in phase was calculated for each single cell passing by for all the frequencies at which measurements were conducted.

### Support vector machine classifier

SVMs are among the best “off-the-shelf” supervised learning algorithms widely used for classification and regression. In other words, when provided a labeled training data set, the algorithm will output an optimal hyperplane that can be used to classify test data. To improve the classification accuracy, we used SVM with a Gaussian Kernel. A kernel function is a form of mapping done to the training data to transform the data in higher dimensions. Mapping data in higher dimensions using a kernel function enables working with highly complex data without significant computational complexity. We used a Gaussian Kernel, which works by calculating the square of the Euclidian distance between two feature vectors. The data used for training consist of features extracted from 100% live and 100% dead cells (based on Vi-Cell data). For training the data, we labeled the features from live cells as 1 and features from dead cells as 0. The training data set size was more than 1000 events (peaks from the impedance data corresponding to a cell passing over the electrodes) to make sure the SVM classifier does not face the problem of overfitting. To evaluate the robustness and accuracy of our SVM classifier, we tested it using three different tumor cell test samples, with differing viability percentages (90% live, 50% live, and 82% live). The number of 1’s predicted by a classifier divided by the total number of samples gave us the viability percentage predicted by the SVM classifier.

### Performance of the SVM classifier

To evaluate the performance of our SVM classifier, we used a confusion matrix on a set of test data for which the true values were known. A part of the training data, which included features from 100% live, and 100% dead cells was used for testing. The confusion matrix was built using this data. The following performance metric was used to evaluate the SVM classifier:

1 $${\rm{Accuracy = (TP + TN)}}/{N}$$

TP (True Positive) is the number of the times the classifier accurately predicted the cell is live, provided the cell was actually alive, TN (True Negative) is the number of times the classifier accurately predicted the cell was dead, provided the cell was actually dead and *N* is the total number of data points.

### Amplitude change as feature for classification

**Fig. 4:**
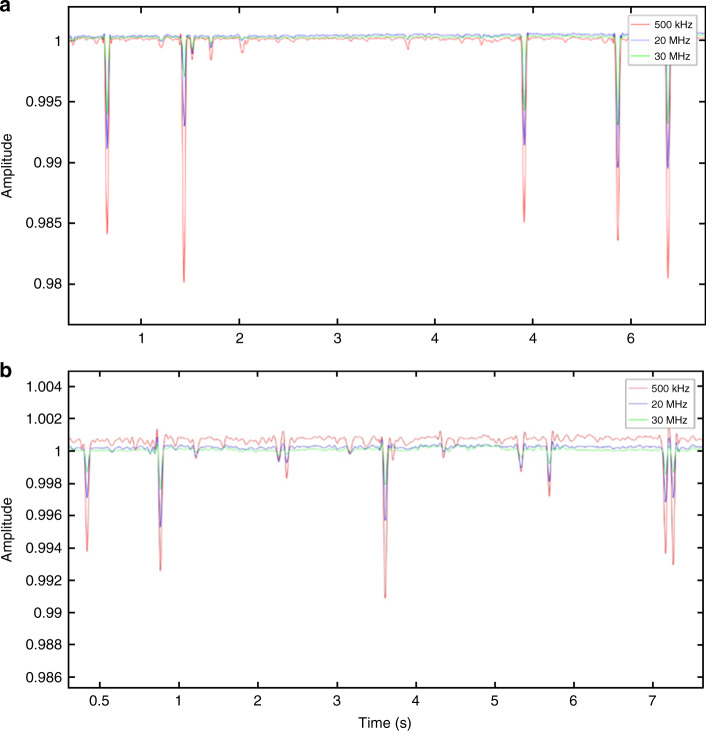
Time series data of single cells. Normalized impedance response of **a** live cancer cells and **b** dead cancer cells at 500 kHz, 20 MHz, and 30 MHz. Each peak corresponds to a single cell being detected

**Fig. 5:**
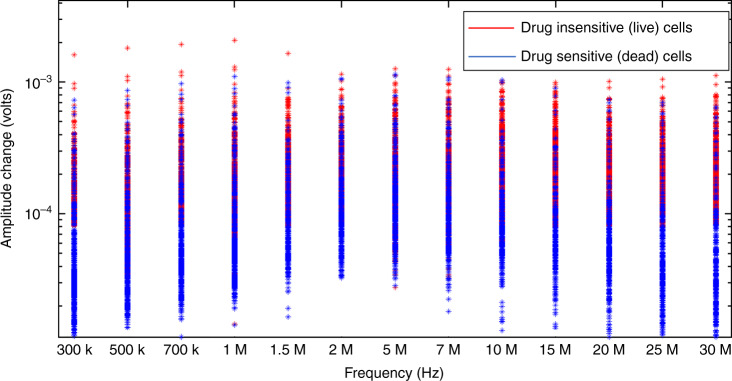
Frequency dependent amplitude change. Amplitude spectrum of live cancer cells and dead cancer cells

**Fig. 6:**
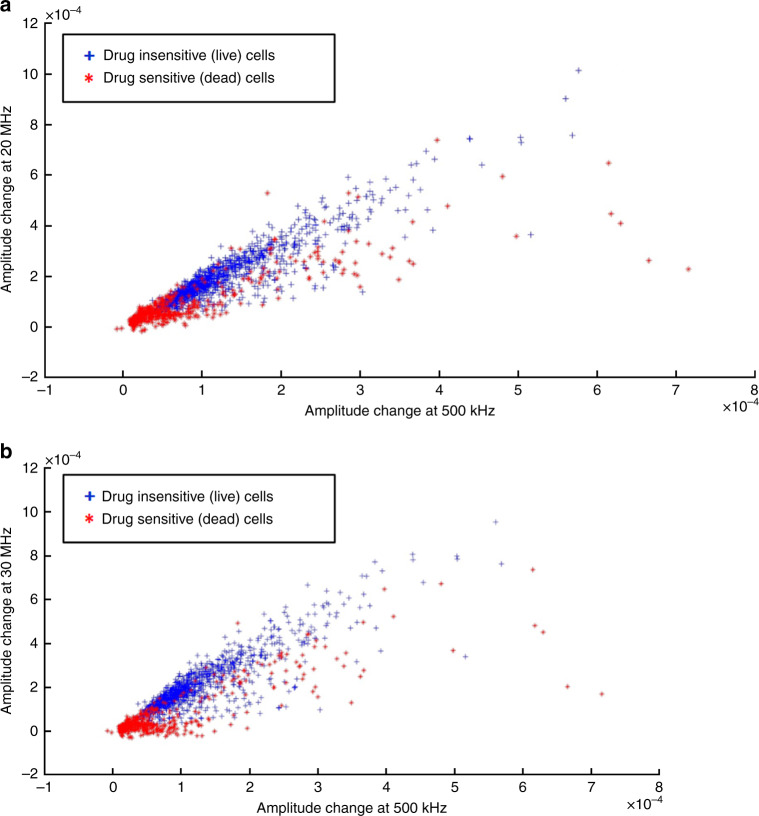
Multi-frequency analysis for amplitude change. **a** Scatter plot of amplitude change for live cancer cells and dead cancer cells at 500 kHz and 20 MHz. **b** Scatter plot of amplitude change for live cancer cells and dead cancer cells at 500 kHz and 30 MHz

**Fig. 7:**
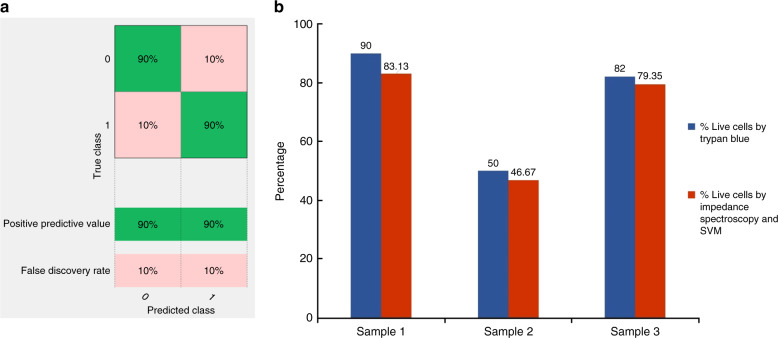
Classifier accuracy when amplitude change used as features. **a** Confusion matrix of the SVM classifier while using amplitude change as features. **b** Comparison between the analysis of cell viability by Trypan Blue staining method (ground truth) and multifrequency impedance spectroscopy with SVM using impedance change as features for the SVM classifier

We studied amplitude change at different frequencies as features for our SVM classifier. Figure 4a, b represents the normalized impedance response of live cancer cells and dead cancer cells (T47D cancer cells) at 500 kHz, 20 MHz, and 30 MHz. Higher frequencies (>10 MHz) probe the internal properties of the cell^21^. Figure 5 presents the spectrum of amplitude change for live and dead cancer cells at a wide range of frequencies. We used amplitude change at 500 kHz, 20 MHz, 25 MHz, and 30 MHz as features for our SVM classifier. Figure 6a, b shows scatter plots representing amplitude change for live cancer cells and dead cancer cells at various frequencies. The confusion matrix for the SVM classifier using amplitude change as the feature is shown in Fig. 7a. Our classifier reported an accuracy of 89.7%, with a TP and TN rate of 90% each. Figure 7b represents a bar graph comparing the cell viability obtained using Trypan Blue staining (ground truth) and multifrequency impedance spectroscopy with SVM using amplitude change as features for the SVM classifier.

### Phase change as feature for the SVM classifier

**Fig. 8:**
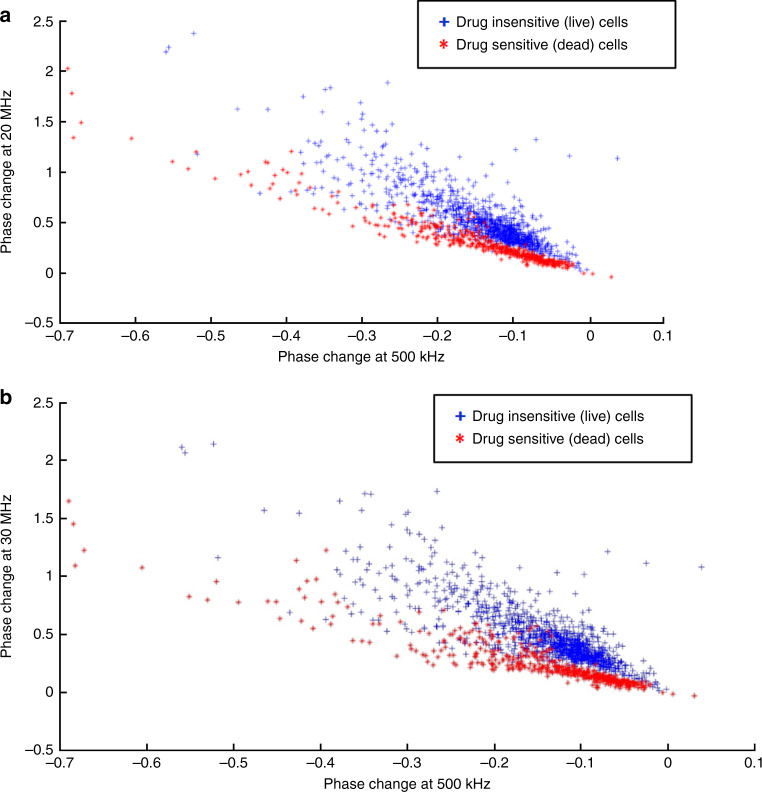
Multi-frequency phase analysis. **a** Scatter plot of phase change for live cancer cells and dead cancer cells at 500 kHz and 20 MHz. **b** Scatter plot of phase change for live cancer cells and dead cancer cells at 500 kHz and 30 MHz

For phase change, we observed a general trend wherein the change (in phase) was negative at lower frequencies (<1MHz) and positive at higher frequencies. Figure 8a, b presents scatter plots representing phase change for live cancer cells and dead cancer cells (T47D cancer cells) at various frequencies. We tested our SVM classifier for all four frequency sets. The frequency set consisting of phase change at 500 kHz, 20 MHz, 25 MHz, and 30 MHz reported higher accuracy for samples containing different percentages of viable cells.

**Fig. 9:**
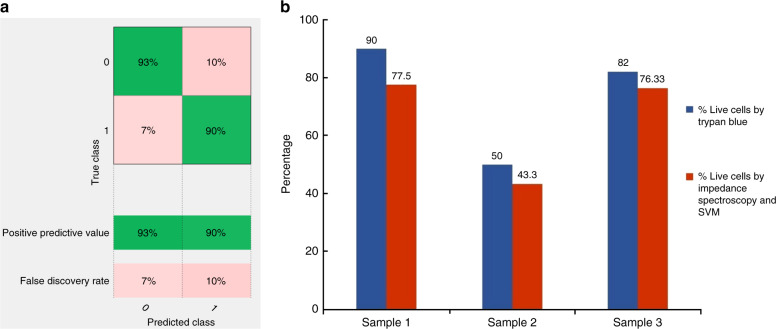
Classification accuracy when phase change used as features. **a** Confusion matrix of the SVM classifier while using phase change as features. **b** Comparison between the analysis of cell viability by Trypan Blue staining method (ground truth) and multifrequency impedance spectroscopy with SVM using phase change as features for the SVM classifier

The confusion matrix for the SVM classifier, using phase change as the feature is shown in Fig. 9a. Our classifier reported to have an accuracy of 90.6%, a TP rate of 90% and a TN rate of 93%. Figure 9b represents a bar graph comparing the analysis of cell viability by Trypan Blue staining method (ground truth) and multifrequency impedance spectroscopy with SVM using phase change as features for the SVM classifier.

### Amplitude change and phase change as features for the SVM classifier

**Fig. 10:**
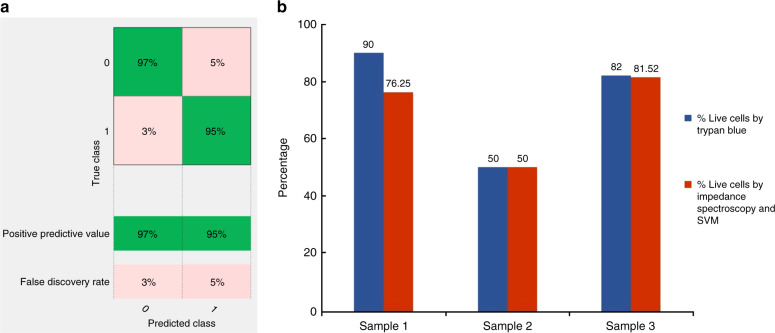
Classification accuracy when both amplitude and phase change used as features. **a** Confusion matrix of the SVM classifier while using amplitude change and phase change as features. **b** Comparison between the analysis of cell viability by Trypan Blue staining method (ground truth) and multifrequency impedance spectroscopy with SVM using amplitude change and phase change as features for the SVM classifier

Lastly, we explored the use of amplitude change and phase change as features for the SVM classifier. Since, for the same frequency sets (500 kHz, 20 MHz, 25 MHz, and 30 MHz) amplitude change and phase change individually gave good results, we built an 8-feature matrix which included both amplitude change and phase change. Before training the 8-feature matrix with the SVM classifier, the data points were normalized to ensure that all the data points lie within a specified range. The confusion matrix for the SVM classifier with amplitude change and phase change as features is shown in Fig. 10a. Our classifier had an accuracy of 95.9%, a TP rate of 95% and a TN rate of 97%. Figure 10b represents a bar graph comparing the analysis of cell viability by Trypan Blue staining (ground truth) and multifrequency impedance spectroscopy with SVM using amplitude change and phase change as features for the SVM classifier. The prediction accuracy for Sample 1 saw no improvement, compared to when only phase or amplitude change was used for training.

## Conclusion

In this work we built a device capable of rapidly analyzing cell viability without staining of cells, which allows for further downstream molecular analysis. We explored the use of phase change at different frequencies as features for the SVM classifier. We found that phase change follows a trend, negative at lower frequencies and positive at higher frequencies, but the quantitative change in phase for different types of cells enables higher accuracy classification. Compared to optical techniques for label-free analysis of cell viability, our novel method can rapidly analyze cell viability with minimal cost. We envision using this device as a point-of-care diagnostic for assessing patient response and personalization of therapeutics. Although beyond the scope of this study, we also consider ultimate practical implementation in the clinical setting. When dealing with patient samples, this drug sensitivity assay can be used for screening dissociated cells obtained from a tumor biopsy. Various protocols and kits are available commercially to dissociate cells into suspension from human tissues and tumors^38^. In case heterogeneity of tumor cells from patient-to-patient ends up being significant, for each test subject, it may be necessary to train the classifier on live cells from the tumor (untreated with drug) and tumor cells killed via heat shock. The trained classifier would then be capable of testing viability of cells incubated with the drug to assess efficacy. Future efforts will be dedicated toward testing with clinical samples.

## Materials and methods

Electrodes are fabricated on glass using standard photolithography on a 3″ fused silica wafer. The process consists of photo-patterning resist on the fused silica wafer, electron beam metal evaporation, and liftoff processing. The process of photo-patterning includes wafer cleaning, spin coating the photoresist, soft bake of the resist, ultraviolet light exposure through a chromium mask printed on a 4″ × 4″ glass plate, resist development, and hard bake of the resist. Following the photo-patterning process, a 100-nm-gold layer is deposited on the substrate using electron beam evaporation. A 10-nm layer of chromium is used to enhance the adhesion of gold to the glass wafer; otherwise the gold film gets peeled off easily. We chose gold as the electrode due to its resistance to corrosion and its inert nature. The width of the electrodes was 20 μm and spacing between the two electrodes was 25 μm.

### Microfluidic channel fabrication

We fabricated the microfluidic channel itself in PDMS (Poly-dimethylsiloxane) by using soft lithography. A layer of SU-8 was patterned onto a 3″ Silicon wafer that acts as a master mold. The SU-8 photo-patterning process involves standard cleaning, spin coating, soft baking, exposure, development, and hard baking. After the master mold was fabricated, PDMS (10:1 prepolymer/curing agent) was poured onto the master mold and baked at 80 °C over 2 h for curing. The PDMS channel was then peeled off from the mold. A 5-mm hole and a 1.5-mm hole were then punched to form the inlet and outlet, respectively. The PDMS substrate was then aligned and bonded to the electrode chip after both substrates have undergone oxygen plasma treatment. The bonded chip was then baked at 70 °C for 30 min to form the irreversible bond. Our microfluidic channel had a width of 100 μm and height of 30 μm. Figure 2a represents microfabricated electrodes bonded with the channel and cancer cells flowing through the microfluidic channel as shown in Fig. 2b.

### Cell culture and cytotoxicity test

T47D breast cancer cell line (in American Tissue Culture Collection also known as-HB-133) is a luminal type-A breast cancer cell line obtained from a pleural effusion from a ductal breast cancer carcinoma patient. The cell line is also classified according to the expression of the receptors for hormonal therapy and thus classified as ER+ (Estrogen receptor positive), PR+ (Progesterone receptor positive), and HER2− (Herceptin receptor 2 negative).

For cell culture, RPMI 1640 media and fetal bovine serum albumin from Invitrogen (Fischer Scientific) were used. For the cytotoxicity assay, 5000 T47D cells per well were plated in RPMI 1640 media (Gibco) supplemented with 10% FBS (Invitrogen). After overnight culture, spent media was removed and fresh media containing drug was added and plates were incubated for 72 h. To assess cell viability of the T47D breast cancer cell line, at the end of the experiment the 3-(4,5-dimethylthiazol-2-yl)-5-(3-carboxymethoxyphenyl)-2-(4-sulfophenyl)-2H-tetrazolium, inner salt (MTS) assay was performed according to the CellTiter 96 Aqueous One Solution protocol (Promega, Madison, WI) according to manufacturer’s protocol.

The cells were collected after the drug incubation time (72 h) and cell viability was determined using the Vi-CELL™ Series Cell Viability Analyzer (Beckman Coulter, Carlsbad, CA). The cytotoxicity data were further analyzed using GraphPad Prism 4 software (GraphPad Software Inc., CA). The 50% inhibitory concentration (IC50; the drug concentration required to obtain 50% cell kill compared to control) was determined using the nonlinear regression curve fit of the graphs drawn by GraphPad Prism 4 software. All experiments were performed in triplicate wells, and all experiments were repeated at least three times. The cells in logarithmic phase of growth were analyzed. T47D cells treated with our antibody-drug conjugate undergoes apoptosis, thus the cell membrane ruptures and modifies the structure of the cell, changing cell shape and diameter analyzed by the Vi-CELL analyzer.

### Microfluidic channel operation

For all experiments, we relied on gravity flow to pump the fluid through the microchannel without relying on syringe pumps; given that syringe and tubing itself often introduces electronic noise and interference into the system as well. As we relied on gravity flow, this required that the channel walls be hydrophilic so that the capillary action would allow the fluid to be wicked easily and thus not impede the movement of fluid once in the channel. PDMS (Poly-dimethylsiloxane) is hydrophobic in nature, thus before every experiment we treated the bonded microchannel with oxygen plasma for 30 s to make the channel hydrophilic. The channel was filled with phosphate buffer saline (PBS) to preserve hydrophilicity. The difference in height resulted in a pressure difference between the inlet and the outlet, which generates flow. All measurements were performed inside a metal box to minimize external interference with the sensor and 60 Hz coupling.
